# Developmental venous anomaly coexisting with arteriovenous malformation: a case report

**DOI:** 10.3389/fsurg.2025.1562013

**Published:** 2025-03-18

**Authors:** Mei Li, JiangBo Ding, XiTao Zong, Chi Lin, QingLing Liu, XiaoPeng Chen, JiaXiong Wang

**Affiliations:** ^1^ Department of General Medicine, South Yunnan Central Hospital of Yunnan Province (The First People’s Hospital of Honghe Prefecture), Mengzi, China; ^2^ Department of Neurosurgery, South Yunnan Central Hospital of Yunnan Province (The First People’s Hospital of Honghe Prefecture), Mengzi, China

**Keywords:** cerebrovascular malformations, developmental venous anomaly, arteriovenous malformation, venous thrombosis, postoperative anticoagulation

## Abstract

We describe a rare case of a developmental venous anomaly associated with an arteriovenous malformation. A 20-year-old male presented with seizures and was diagnosed with left parietal arteriovenous malformation combined with developmental venous anomaly in the left frontal lobe, with the draining veins of both lesions converging into the same bridging vein despite the lesions affecting anatomically distinct areas. The patient underwent a craniotomy for resection of the arteriovenous malformation. However, progressive aphasia developed on the third postoperative day. Subsequent neuroimaging (CT and MRI) revealed thrombosis formation within the drainage vein of the developmental venous anomaly. The symptoms of aphasia gradually disappeared after anticoagulant therapy with low molecular weight heparin. This case adds to the current consensus that developmental venous anomalies have normal venous drainage. It also suggests that developmental venous anomalies are susceptible to hemodynamic changes.

## Introduction

Arteriovenous malformation (AVM), capillary telangiectasia, cavernous malformation, and developmental venous anomaly (DVA) represent 4 commonly recognized subgroups of cerebrovascular malformations (CVMs) (1). There is also the possibility of coexistence of two or more CVMs in the same patient or within the same lesion. When two cerebrovascular diseases occur in the same patient, the harm of the disease and the complexity of treatment often increase. According to the literature, association of DVA with cavernous malformation is most common, occurring at a rate of 13%–40%, but association of DVA and AVM is very rare and only few cases are reported (2–4). Unlike cavernous malformations, AVMs can significantly affect hemodynamics. Therefore, there is still a lack of experience in how to treat developmental venous anomalies coexisting with cerebral arteriovenous malformations. Here we present our experience in the treatment of a special case of developmental venous anomaly associated with cerebral arteriovenous malformation.

## Case report

**Figure 1:**
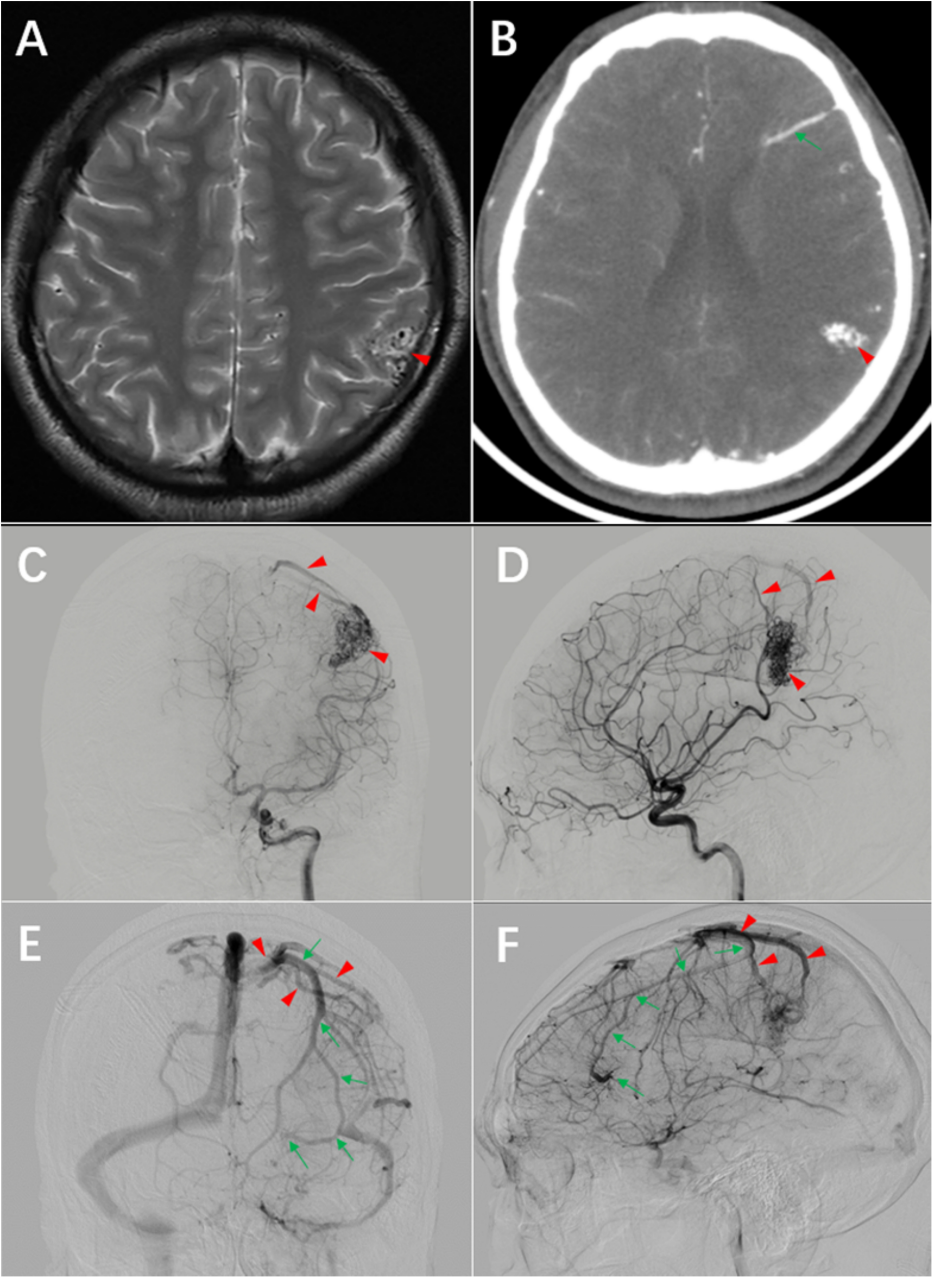
Preoperative imaging. **(A)** T2-weighted MRI demonstrates a lesion adjacent to the postcentral gyrus of the left parietal lobe (arrowhead). **(B)** CTA shows a disorganized vascular network in the left parietal lobe (arrowhead) and linear vascular structures in the frontal lobe (arrowhead). **(C)** and **(D)** DSA arterial phase reveals the arteriovenous malformation (AVM) and its draining vein (arrowhead). **(E)** and **(F)** DSA venous phase displays the DVA with its draining vein (arrow), which converges with the AVM's draining vein (arrowhead).

A 20-year-old male right-handed patient presented with a one-year history of unprovoked recurrent seizure episodes characterized by focal onset evolving to bilateral tonic-clonic convulsions. Typical manifestations included transient dizziness preceding the seizures, initial focal motor involvement in the right upper extremity, secondary generalization leading to whole-body convulsions, associated loss of consciousness and oral frothing. Each episode lasted 2–5 min and resolved spontaneously without residual neurological deficits. The seizures occurred 1–2 times per month without identifiable triggers. There were no cluster episodes or status epilepticus, and the patient did not experience cephalgia, sensory disturbances, or motor impairments between events. The left parietal arteriovenous malformation was diagnosed by MRI and cerebral angiography at another hospital one year ago. The patient was worried about the risk of surgery and did not undergo surgery. He did not take antiepileptic drugs regularly and had recurrent seizure episodes for one year. He was admitted this time for surgical treatment. On physical examination, the blood pressure was 118/79 mmHg, and the GCS score was 15. The general condition was good, and the comprehension, memory, orientation and calculation ability were normal. Power in upper and lower limbs were grade 5, sensations were intact bilaterally in upper and lower limbs. Magnetic resonance imaging showed that the lesion was located in the left parietal lobe, adjacent to the postcentral gyrus, CTA showed disordered vascular mass in the left parietal lobe and a straight vessel in the left frontal lobe. The DSA confirmed an AVM in the left parietal lobe and a DVA in the left frontal lobe. Both lesions shared a common draining vein, ultimately draining into the superior sagittal sinus. The AVM was visualized on CTA as a conical structure with its apex directed toward the deep brain, measuring 2.8 cm × 1.7 cm × 1.2 cm. During the arterial phase of DSA (Digital Subtraction Angiography), a densely packed arteriovenous malformation (AVM) is observed in the left parietal lobe. A branch from the M4 segment of the middle cerebral artery enters the nidus anteriorly, demonstrating terminal direct supply with marked compensatory dilation of this feeding artery. Two superficial draining veins are visualized superior to the nidus, draining into the superior sagittal sinus. A stenosis is noted in the posteriorly located draining vein. No deep draining veins are identified. In the late venous phase of the DSA, a DVA is observed in the left frontal lobe, demonstrating a “caput medusae” appearance. Its draining vein follows an extended course before converging with the draining vein of the AVM (Figure 1).

**Figure 2:**
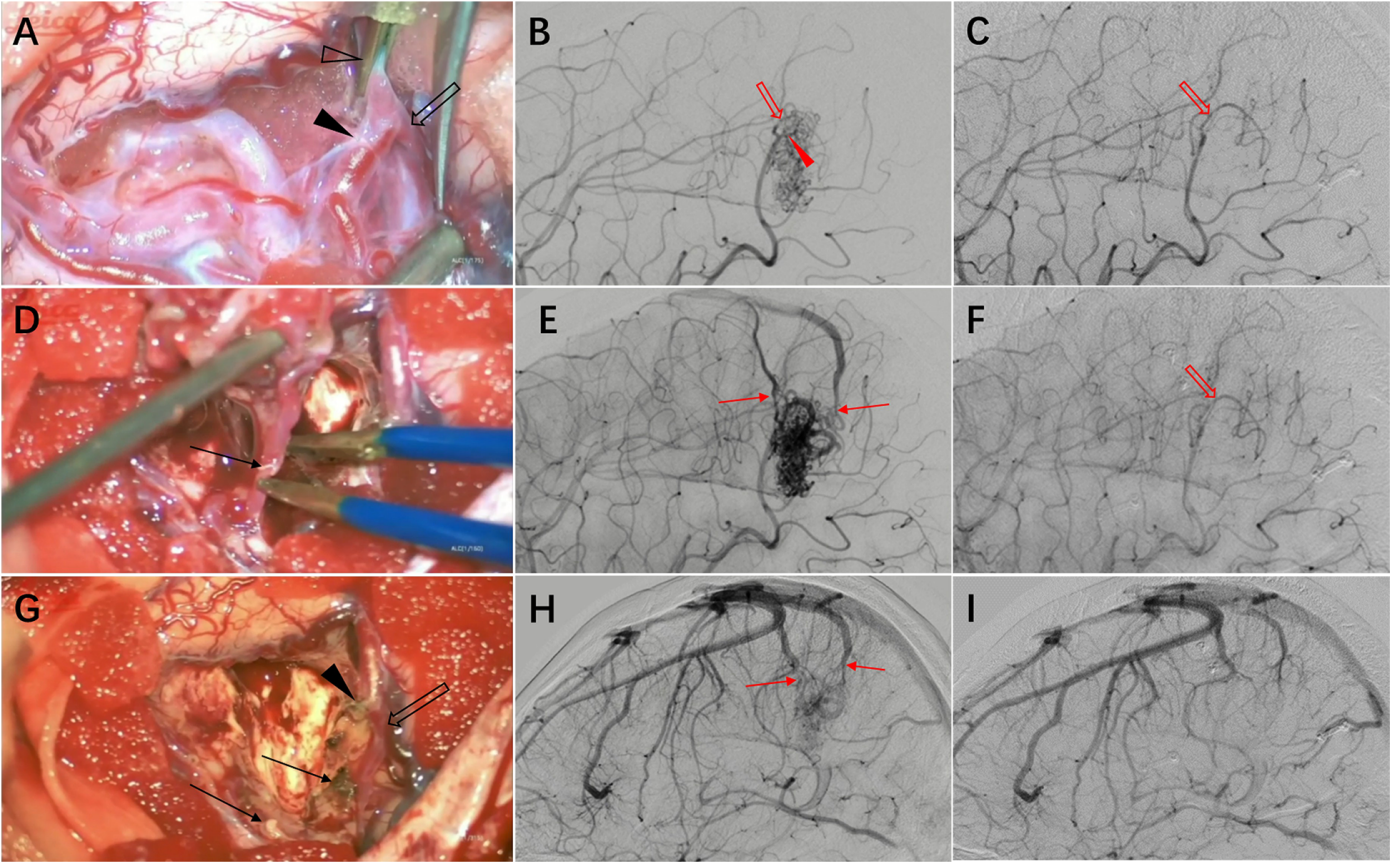
Intraoperative findings and imaging correlation. **(A)** Intraoperative management of the feeding artery side of the malformation vascular cluster. A temporary clip (hollow arrowhead) was applied near the AVM nidus to occlude the feeding artery. Anatomical dissection revealed a distal bifurcated vessel with one branch as the feeding artery (solid arrowhead) and the other supplying normal brain tissue (hollow arrow), which could not be differentiated by imaging. **(B)** Preoperative DSA demonstrates the feeding artery and AVM nidus. **(C)** Postoperative DSA shows the preserved normal brain tissue feeding artery (hollow arrow) identified intraoperatively. **(D)** The draining vein (arrow) was preserved until the final stage of AVM resection, then coagulated and divided. **(E)** Preoperative DSA displays the feeding artery, AVM nidus, and draining vein. **(F)** Postoperative DSA demonstrates complete resection of the AVM nidus with absence of premature venous drainage. **(G)** Post-resection view showing the proximal stump of the main feeding artery (solid arrowhead), preserved normal brain tissue feeding artery (hollow arrow), and draining vein stump (arrow). **(H)** Preoperative DSA reveals the draining vein and adjacent normal veins. **(I)** Postoperative DSA confirms preservation of normal venous structures.

The neurosurgeon and the neurointerventionist jointly evaluated the treatment plan, given that the AVM had a Spetzler-Martin grade of 2, was superficially located, and was considered suitable for craniotomy. However, interventional embolization carries a higher risk of venous thrombosis, which is more likely to involve the DVA. Understanding the architectural features of the AVM is crucial for formulating an appropriate surgical strategy. This is a superficial compact-type AVM that can be dissected along the interface between the AVM nidus and the surrounding brain tissue. The main feeding artery is located superficially, which allows for early occlusion to reduce tension within the nidus. This facilitates easier dissection along the boundary between the AVM nidus and normal brain tissue. A craniotomy is performed directly over the AVM nidus. After exposing the lesion, the arachnoid membrane is carefully incised along the lesion's margins. The dissection proceeds in a spiral pattern along the boundary toward the conical apex, with particular attention to clarifying vascular architecture on the arterial supply side. Only confirmed feeding arteries entering the nidus are divided to preserve normal blood supply to the postcentral gyrus. Following interruption of major feeding arteries, attention is directed to the venous drainage side. At this stage, with reduced tension in the AVM nidus, separation from adjacent transiting normal veins becomes more manageable. Larger vessels are temporarily occluded for observation, if no AVM distension occurs, they are coagulated and divided. The draining vein is preserved until final stages when it is coagulated and severed. Complete resection of the AVM is achieved while successfully preserving the DVA. No brain swelling was observed after AVM resection (Figure 2).

**Figure 3:**
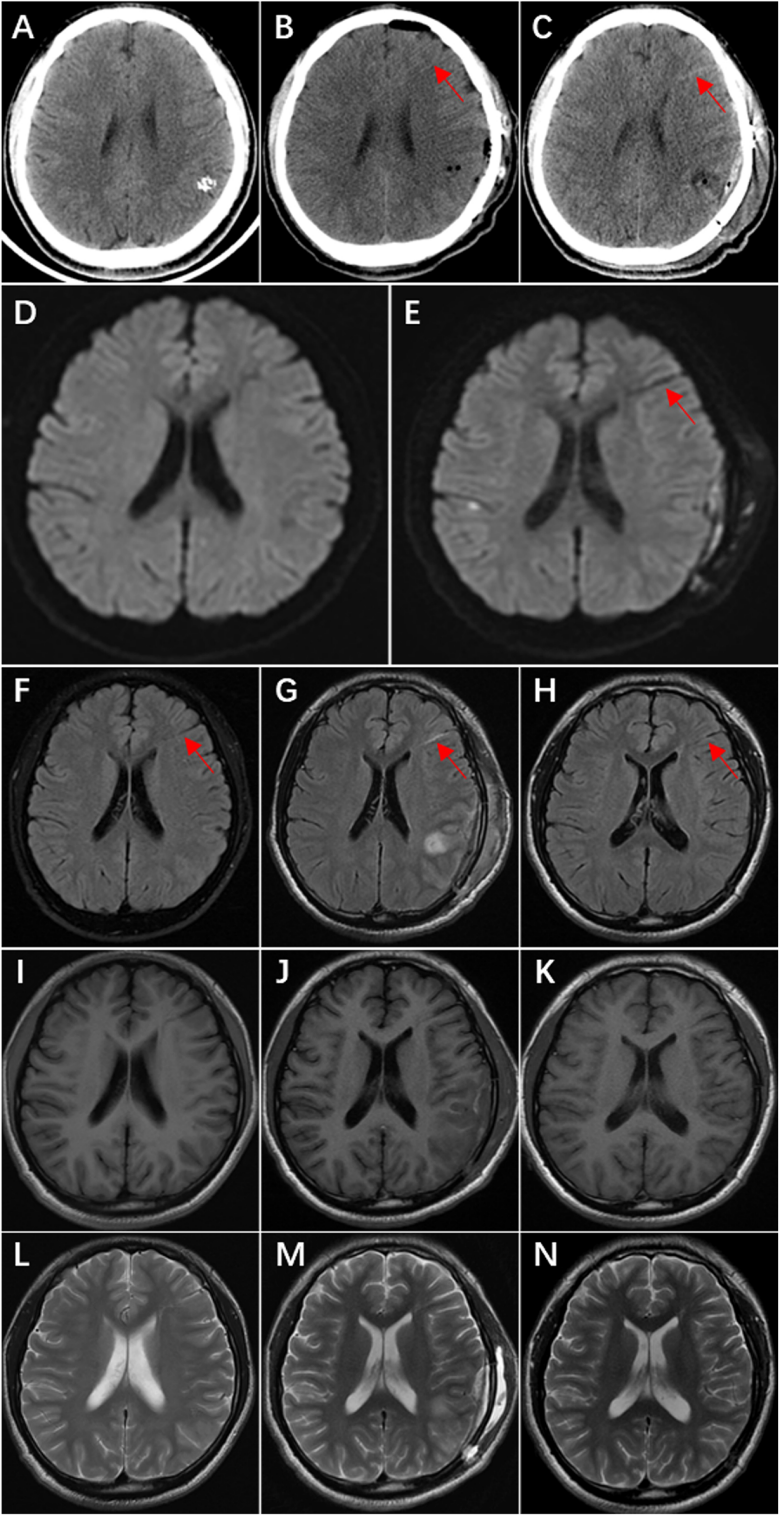
Ct, DWI, and MRI T2 FLAIR demonstrate postoperative thrombosis within the draining vein of the DVA. **(A)** Preoperative CT shows no hyperdense area in the left frontal lobe. **(B)** Postoperative Day 1 CT reveals a linear slightly hyperdense lesion (arrow) in the left frontal lobe. **(C)** Postoperative Day 4 CT shows increased prominence of the linear hyperdense area (arrow) in the left frontal lobe. **(D)** Preoperative DWI demonstrates no obvious linear hypointense signal in the left frontal lobe. **(E)** Postoperative Day 5 DWI displays a distinct linear hypointense signal (arrow) in the left frontal lobe. **(F)** Preoperative MRI T2 FLAIR exhibits a linear hypointense area (arrow) in the left frontal lobe. **(G)** Postoperative Day 5 MRI T2 FLAIR shows transformation of the linear hypointense signal into hyperintensity (arrow). **(H)** Follow-up examination at 4 months postoperatively reveals reversion to hypointensity (arrow). I–N. MRI T1 and T2 sequences show no significant signal changes in the left frontal lobe: I & L (preoperative), J & M (postoperative Day 5), K & N (4 months postoperatively).

**Figure 4:**
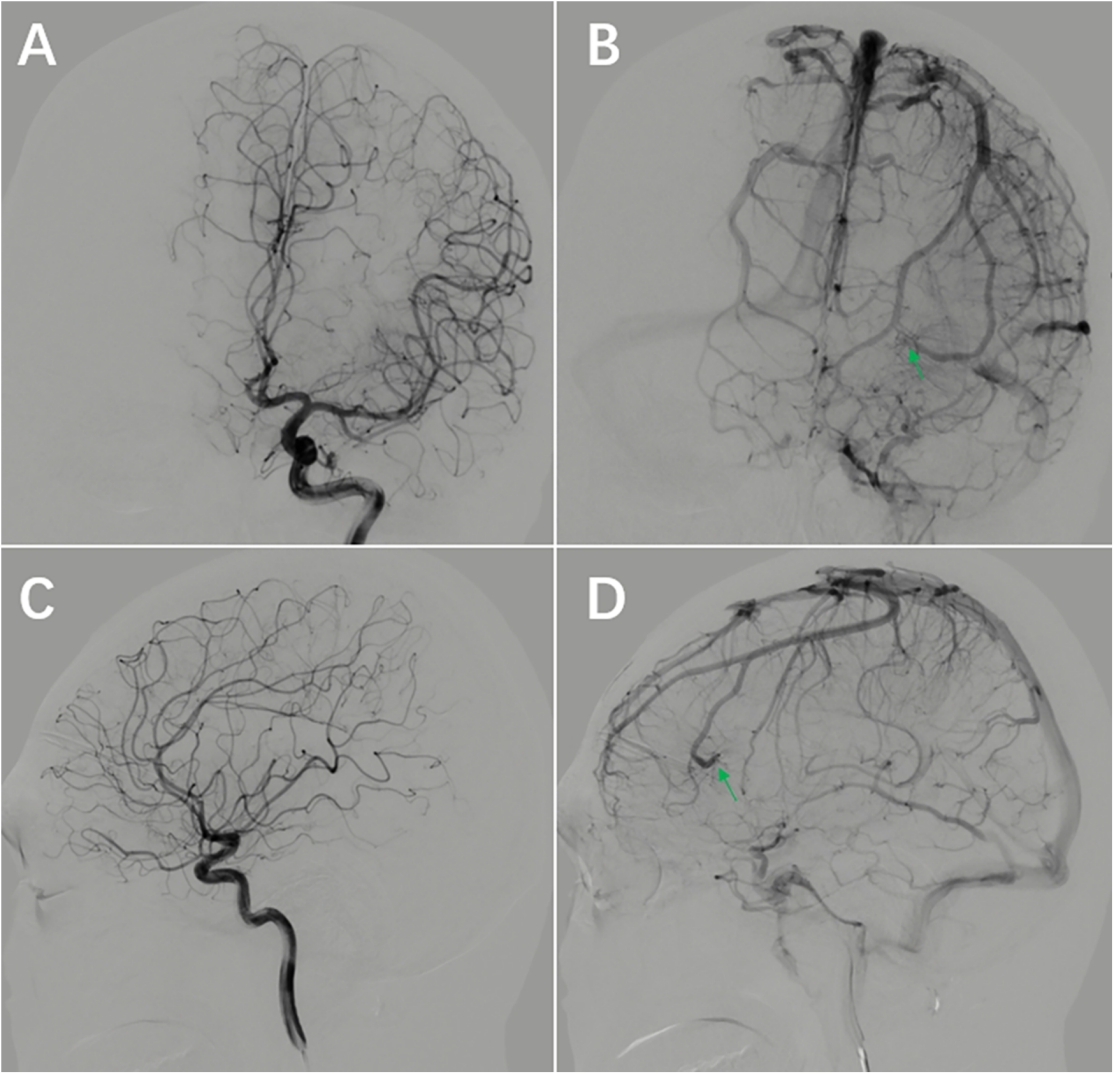
Follow-up DSA at 7 months postoperatively. **(A–D)** The AVM is not visualized DVA remains unchanged compared to preoperative imaging (arrow).

On the first day after surgery, the patient did not have any neurologic symptoms, CT scan showed a linear slightly hyperdense area in the left frontal lobe, but this anomaly was ignored because it was too small. On the fourth day after surgery, the patient developed transient aphasia, CT scan showed the linear hyperdense area of the left frontal lobe was more obvious, and it was ignored again. On the fifth day after surgery, the patient had frequent episodes of aphasia, MRI showed linear abnormal signals in the left frontal lobe, and the linear abnormal signal is consistent with the location of linear hyperdense area on CT (Figure 3). This suggests venous thrombosis in the DVA, and the symptoms of aphasia disappeared completely after anticoagulant therapy with nadroparin. During the follow-up of 7 months, the patient took sodium valproate regularly and had no recurrence of epilepsy. There were no neurologic deficits. The DSA reexamination revealed no recurrence of AVM, and the DVA remained unchanged post-operation (Figure 4).

## Discussion

Cerebral developmental venous anomaly (DVA) is widely used as a synonym for venous angioma, cerebral venous malformation, or cerebral venous medullary malformation. DVAs are the most frequently encountered cerebral vascular malformations, with an incidence of up to 2.6% in a series of 4,069 brain autopsies (5). Clinically, DVAs are usually discovered incidentally with an overall incidence of 2%–4% (1), the estimated risk of hemorrhagic strokes in an isolated DVA is 0.15%–0.68% (6). The incidence of AVM is generally cited as around 1 in 100,000, and the annual hemorrhage rate of an AVM is 2%–3% (3). Co-occurrence of a DVA and an AVM is rare but has a higher bleeding risk than AVM alone (69% vs. 38%) (7).

In this patient, the AVM was located in the left parietal lobe, and the coexisting developmental venous anomaly was located in the left frontal lobe. The draining veins of these two lesions converge into the same draining vein. Such a relationship helps to observe the functional changes of the DVA before and after surgery. Postoperative CT showed that the drainage vein of DVA became high-density, which was the imaging feature of thrombosis (5), MRI also showed significant changes before and after surgery, and the aphasia symptom disappeared after anticoagulant therapy. It can be inferred that the patient had DVA drainage vein thrombosis after operation. This case provides strong evidence for several points.

First, DVAs have normal drainage functions, and injury may lead to neurological deficits. The etiology and pathogenesis of DVAs are widely attributed to either arrested development of normal parenchymal veins or occlusion of developing medullary veins, with persistent embryonic venous channels serving as a compensatory drainage system (4). In a retrospective study, the natural history of venous angiomas was followed up over a 14-year period in 100 patients with radiographically identifiable lesions and the complications that could possibly be related to the lesion were hemorrhage in one patient, seizures in five, and transient focal deficits in eight. The authors concluded that significant complications secondary to venous angiomas are infrequent (8). Removal of DVAs can be complicated by brain swelling and hemorrhagic infarction and even death (9, 10). In this case, the AVM was situated distant from Broca's area, whereas the DVA occupied an adjacent anatomical position to this critical speech center. Although the patient maintained intact language function preoperatively, postoperative thrombosis of the DVA led to the onset of aphasia. This clinical evidence substantiates that DVAs serve essential drainage functions and should be preserved. DVAs may also coexist with or be associated with extraaxial developmental venous anomalies (eDVAs). When surgical intervention is indicated for an eDVA, it remains crucial to differentiate it from intraaxial DVAs or to exclude the coexistence of intraaxial DVAs (11).

Second, DVAs lack autoregulatory capacity and are susceptible to hemodynamic changes. DVAs are composed of dilated medullary veins converging centripetally into a large collecting venous system, they can have potential venous hypertension and can be vulnerable to hemodynamic changes. Histological examination found DVAs had dilated thin-walled vessels that were diffusely distributed in the normal white matter (9). Masson et al. (12) suggested that DVAs lack smooth muscle cells and elastic connective tissue, have limited ability to regulate and adapt, and are prone to thrombosis when hemodynamics change. In this case, removal of the AVM would not have caused mechanical harassment of the DVA, but because the draining veins of the two lesions joined together, the hemodynamics change after removal of the VAM would have directly affected the DVA, and led to thrombosis.

Third, anticoagulation therapy should be considered when there are hemodynamics change that may affect DVAs. In the treatment of arteriovenous fistulas, venous thrombosis may occur when blood flow slows down in the dilated high-flow draining vein (13). A large single-center experience found that it's necessary to use anticoagulant or antiplatelet therapy to prevent cerebral venous thrombosis after resection of AVM with extensive venous outflow network (14). The patient will also have blood stasis in the draining vein after AVM resection, which will cause venous thrombosis. The linear hyperdensity on CT and linear abnormal signals on DWI, as well as the disappearance of symptoms after anticoagulant therapy with low molecular weight heparin, confirmed the presence of venous thrombosis within the DVA after AVM resection in this patient.

In conclusion, DAV coexisting with AVM is a rare condition. It is particularly important to maintain patency of the DVA at all times during treatment. Developmental venous anomalies have normal drainage function, but are more likely to become dysfunctional when affected by hemodynamic changes than normal veins. Therefore, more aggressive consideration of anticoagulation may be required under certain conditions. In addition, hyperdense signal seen on post operative CT in the region of a preexisting DVA should raise the possibility of thrombosis of the DVA in the minds of the surgeon, as this may help in starting anticoagulation early and preventing devastating complications.

## Data Availability

The original contributions presented in the study are included in the article/Supplementary Material, further inquiries can be directed to the corresponding author.
